# RIOT—Rapid Immunoglobulin Overview Tool—annotation of nucleotide and amino acid immunoglobulin sequences using an open germline database

**DOI:** 10.1093/bib/bbae632

**Published:** 2024-12-02

**Authors:** Paweł Dudzic, Bartosz Janusz, Tadeusz Satława, Dawid Chomicz, Tomasz Gawłowski, Rafał Grabowski, Przemek Jóźwiak, Mateusz Tarkowski, Maciej Mycielski, Sonia Wróbel, Konrad Krawczyk

**Affiliations:** NaturalAntibody S.A., Al. Piastów 22, 71-064 Szczecin, Poland; NaturalAntibody S.A., Al. Piastów 22, 71-064 Szczecin, Poland; NaturalAntibody S.A., Al. Piastów 22, 71-064 Szczecin, Poland; NaturalAntibody S.A., Al. Piastów 22, 71-064 Szczecin, Poland; NaturalAntibody S.A., Al. Piastów 22, 71-064 Szczecin, Poland; NaturalAntibody S.A., Al. Piastów 22, 71-064 Szczecin, Poland; NaturalAntibody S.A., Al. Piastów 22, 71-064 Szczecin, Poland; NaturalAntibody S.A., Al. Piastów 22, 71-064 Szczecin, Poland; NaturalAntibody S.A., Al. Piastów 22, 71-064 Szczecin, Poland; NaturalAntibody S.A., Al. Piastów 22, 71-064 Szczecin, Poland; NaturalAntibody S.A., Al. Piastów 22, 71-064 Szczecin, Poland

**Keywords:** immunoinformatics, biologics, antibody numbering

## Abstract

Antibodies are a cornerstone of the immune system, playing a pivotal role in identifying and neutralizing infections caused by bacteria, viruses, and other pathogens. Understanding their structure, and function, can provide insights into both the body’s natural defenses and the principles behind many therapeutic interventions, including vaccines and antibody-based drugs. The analysis and annotation of antibody sequences, including the identification of variable, diversity, joining, and constant genes, as well as the delineation of framework regions and complementarity-determining regions, is essential for understanding their structure and function. Currently analyzing large volumes of antibody sequences is routine in antibody discovery, requiring fast and accurate tools. While there are existing tools designed for the annotation and numbering of antibody sequences, they often have limitations such as being restricted to either nucleotide or amino acid sequences; slow execution times; or reliance on germline databases that are closed, frequently changed, or have sparse coverage for some species. Here, we present the Rapid Immunoglobulin Overview Tool (RIOT), a novel open-source solution for antibody numbering that addresses these shortcomings. RIOT handles nucleotide and amino acid sequence processing, comes integrated with an Open Germline Receptor Database, and is computationally efficient. We hope that the tool will facilitate rapid annotation of antibody sequencing outputs for the benefit of understanding antibody biology and discovering novel therapeutics.

## Introduction

Antibody diversity and specificity are crucial for the immune system’s ability to recognize and combat a wide range of pathogens, leading to effective acquired immunity. Humans can produce a multitude of unique antibodies through V(D)J recombination, a process specific to B-cell and T-cell gene segments [1]. Somatic hypermutation further diversifies these antibodies, optimizing their affinity for antigens [2]. Antibodies are highly specific due to the unique antigen-binding sites formed by the variable regions. This specificity is exploited in monoclonal antibodies [3], which are identical antibodies from a single B-cell clone used in medical treatments for diseases such as cancer or autoimmune disorders. The complexity and diversity of antibody repertoires, underpinned by the mechanisms of V(D)J recombination and somatic hypermutation, necessitate sophisticated tools for their analysis and interpretation. Analyzing these repertoires requires several steps, including accurate germline allele identification, precise sequence numbering according to the chosen scheme, and junction analysis. While various tools exist to address these aspects, many are limited by their specialization in specific areas, such as sequence numbering, and often restrict germline assignment to the gene level, lacking the granularity of allele-level assignment. In contrast, RIOT offers a comprehensive solution by providing accurate numbering and precise germline assignment at the allele level. This versatility makes RIOT a significant landmark in the landscape of antibody annotation software.

Antibody numbering schemes, such as the Kabat [4], Chothia [5], Martin [6] and ImMunoGeneTics (IMGT) [7] and others [8, 9], provide a standardized framework for annotating the amino acid positions in antibody sequences. This standardization is essential for comparing antibodies, correct delineation of complementarity-determining regions (CDRs), humanization, understanding their structure–function relationships, and multiple other applications. The numbering schemes are not only fundamental for academic research but also play a crucial role in the development of monoclonal antibodies for therapeutic use.

The widely recognized methods for antibody numbering predominantly fall into two categories: those that rely on sequence alignment in the traditional sense and those based on hidden Markov model (HMM) alignments [10]. Both approaches necessitate a substantial repository of previously annotated antibody sequences to construct the necessary reference databases. Sequence-based methods can perform alignments of input sequences either to pre-annotated antibody sequences or to germline genes. While the reliance on pre-annotated sequences yields effective results for recognized patterns, it encounters limitations with atypical ones.

An example of profile-based numbering, which applies higher significance to conserved regions, was introduced in AbNum [6]. Here, authors assign framework-derived profile segments to input sequence segments. Those are then used to determine region boundaries, and, finally, region sequences are aligned to consensus patterns. While the authors thoroughly investigated the correctness of the numbering results, the method does not provide germline assignment.

Sequence-alignment-based numbering methods offer simplicity and accuracy for well-represented sequences but require a good-quality input germline database. For example, IgBLAST [11], which aligns input sequences to germline alleles to determine regions in the input sequence, achieves good germline assignment accuracy, yet it is computationally expensive and has a limited number of supported numbering schemes. In contrast, HMM-based methods provide robustness and adaptability for unseen sequences at the cost of even higher complexity and computational expense. To generate HMM profiles used as a reference database, scheme-gapped pairs of V and J genes can be used as it was implemented in ANARCI: Antigen receptor Numbering And Receptor ClassificatIon. [12]. This approach, however, yields alignment penalization of unusually long CDRs. By doing alignment of the input sequence to the profile constructed from aligned V-J pairs, the alignment score suffers the more there is a length difference between the sequence and profile due to the gap penalties. This can result in shorter alignments (which do not cover the entire query sequence) being preferred and having better alignment scores than alignments that cover the whole sequence. This issue is illustrated in Fig. 2, where ANARCI fails to number correctly due to this limitation.

Another sequence-based numbering that addressed the robustness problem was implemented in AbRSA [13]. In their approach, authors prepared numbered antibody consensus sequences of heavy and light chains, where each position in the consensus sequence is a list of the most popular antibody residues constructed from the database abYsis [14]. The query sequence is aligned to the consensus sequence using a modified Needleman–Wunch algorithm that considers the consensus residue region when calculating the alignment score. While this approach correctly assigns lower significance to hypervariable regions, it is limited by the number of available numbering schemes, and it does not provide germline assignment.

Further speed improvement with respect to AbRSA was taken by AntPack [15]. The method is the fastest currently; however, it only handles amino acids, and germline genes are assigned using sequence identity rather than obtained by more accurate alignments.

The available tools either focus purely on the annotation of amino acids or nucleotide sequences and are dependent on the underlying database. IMGT and efforts by the Adaptive Immune Receptor Repertoire (AIRR) community continue to be the main sources of germline genes [16–19]. Nonetheless, these undergo frequent updates that can lead to inconsistencies between annotations employing a particular version of each database. Employing different pieces of software for annotation and different germlines results in inconsistent annotations [20]. Standardization of the annotation protocols across nucleotides/amino acids, as well as the underlying germline database, would increase consistencies between analyses.

Here, we present RIOT, software that facilitates consistent annotation for nucleotide and amino-acid immunoglobulin sequences with an integrated Open Germline database with human and mouse sequences. We benchmark RIOT against leading tools in nucleotide sequence annotation and amino-acid sequence annotation showing a best-in-class versatility and a significant decrease in annotation time. We hope that our software will help in the analysis of antibodies by offering a single solution for sequence analysis with a standardized reference germline database.

## Materials and methods

### Annotation pipeline

Here, we present RIOT—Rapid Immunoglobulin Overview Tool—sequence-based antibody numbering software that facilitates fast execution times, accurate germline assignments, and versatile selection of numbering schemes.

**Figure 1:**
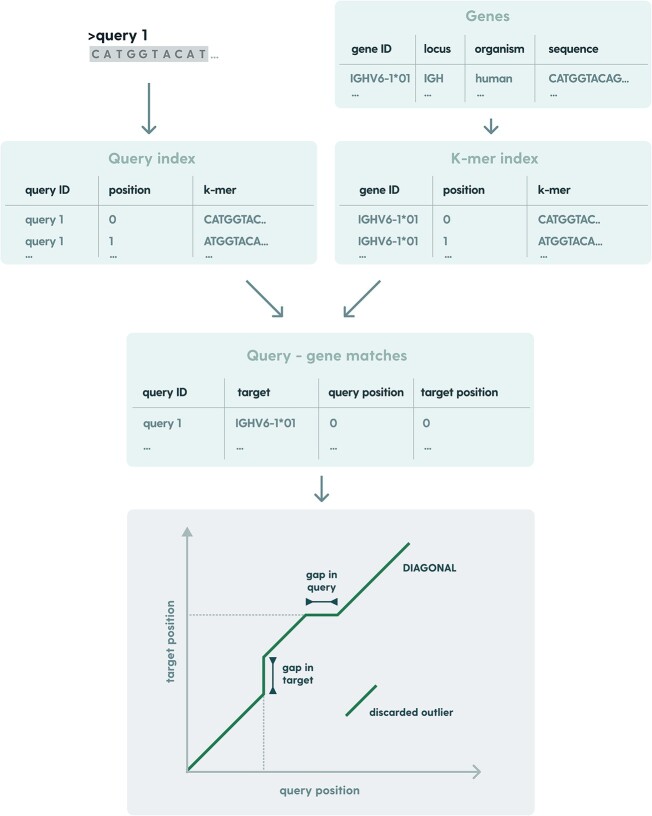
Prefiltering: diagonal score–based candidate alleles selection. Representation of diagonal score calculated for single pair of input query and allele.

At first, V allele candidates are selected in *k*-mer matching–based prefiltering process, and the top 12 matching alleles are pairwise-aligned using the striped Smith–Waterman algorithm [21] to the input sequence in forward- and reverse-complemented directions. The allele with the best e-value is assigned to the sequence together with the source locus and organism. Then, the part of the input sequence that was aligned is masked so the J allele can be identified on the remaining part of the sequence. Only J alleles that match the assigned organism and locus are considered. After further masking the part of the sequence that is aligned to the J allele, the remaining parts of the sequence are used to identify the C allele and in the case of heavy chains—the D allele (Fig. 1). For nucleotide sequences on the input, the reading frame is inferred from V allele alignment, so the input sequence is translated to the amino acid sequence, which is used to align the input sequence to the scheme of choice. After numbering, additional validations are being performed.

### Scheme mapping/numbering

**Figure 2:**
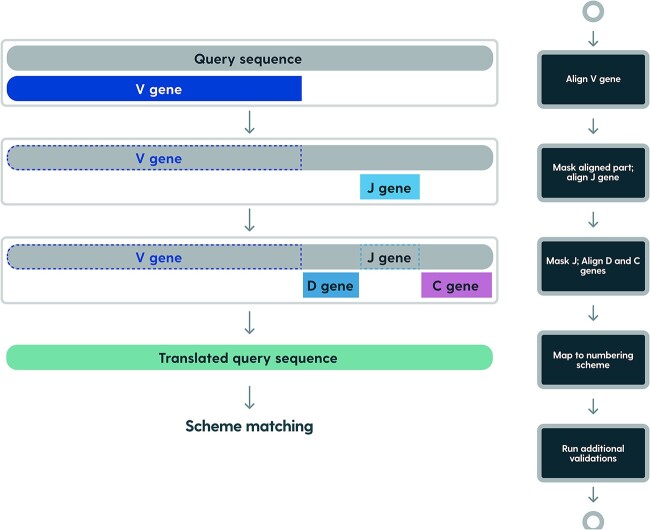
Annotation pipeline: align the query sequence to V alleles (both forward and reverse complemented). Infer the organism, locus, and reading frame from the best V allele alignment. Mask the query sequence aligned with the V allele. Align the J allele, offsetting the best alignment by the masked V region. Mask again the query sequence, and align D and C alleles. Translate query sequence to amino acids using the inferred reading frame. Translate amino acid alignments to a selected numbering scheme (Kabat, Chothia, Martin, or IMGT). The pipeline is identical for nucleotides and amino acids with the exception of a lack of D/C allele annotation in amino acids.

In contrast to the majority of numbering tools, RIOT does not align query sequences to full-length scheme-gapped sequences or profiles. Rather, it aligns V and J segments of the query sequence separately, to ungapped (nonscheme-aligned) germline sequences. Query-germline alignments are then merged with already-known germline-scheme alignments to produce query-scheme mapping on corresponding segments. This approach renders reliable germline assignments and avoids penalization of unusually long CDRs, which is common in artificial sequences deposited in patents [22] (Fig. 2).

We argue that alignment to ungapped allele sequences will yield more accurate germline assignments as we are aligning input to biologically accurate genes rather than sequences where artificially introduced gaps can change alignment scores because of the gap penalties. As a consequence of this, a good-quality germline database is needed on input to provide reliable numbering.

**Figure 3:**
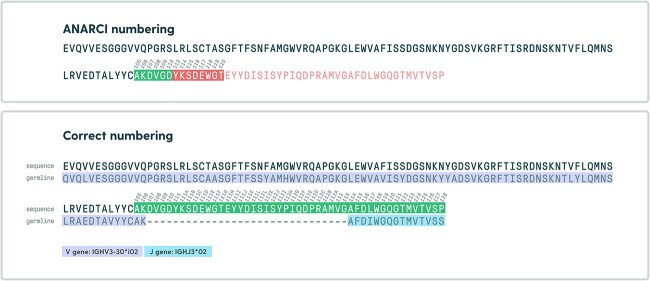
Example of an issue with alignment to artificially gapped sequences. Alignment to IMGT gapped profiles created from V-J gene pairs leads to penalization of unusually long CDR (PDB: 4ocr). To address this, RIOT aligns V and J genes separately. The example above was generated using ANARCI adapted from [23].

We know how germline amino acid sequences map to schemes; therefore, we are able to efficiently combine query-germline alignment with germline-scheme alignment to align input to the scheme of choice. This mapping process is performed by the sequence of steps given in Fig. 3.

Scheme mapping is inferred from the amino acid alignments of the query sequence to V and J alleles. In the case of amino acid sequences on the input, a full allele matching pipeline (prefiltering and pairwise alignments) is executed against amino acid sequences. When nucleotides are provided on the input, the query sequence is matched to V, D, J, and C nucleotide alleles, and the translated query sequence is further pairwise aligned to amino acid sequences of V and J alleles identified from nucleotide allele matching. Then, query-germline alignments are merged with germline-scheme alignments to produce query-scheme mapping for V- and J-derived fragments of the input sequence. The merging is executed by following query-germline and germline-scheme alignment backtraces. This way, produced query-scheme alignments for V and J segments are then collapsed so that all consecutive insertions and deletions are converted to matches. In the next step, V and J query-scheme alignments are merged together, so full query-target scheme alignment is generated, which allows to determine region boundaries. To infer the scheme mapping between V and J alignments, the difference between scheme legal positions and the length of the actual unaligned query sequence segment is calculated. If the query fragment between V and J alignments is longer than the number of legal scheme positions, the segment alignment consists of the number of matches that are allowed in the scheme with additional insertions put in the middle. Similarly, if the query sequence is shorter, the middle segment consists of matches in count equal to the length of the sequence, and missing scheme positions are filled as deletions in the middle. Finally, CDR regions are renumbered so that they match scheme definitions—insertions are put after the specified position (or in the middle for the IMGT scheme) and deletions are put before the specified position. If there are too many deletions to be put before the specified position, they are appended after the specified place.

Framework and CDR boundaries are inferred from query sequence-scheme alignment using the definitions provided by Andrew Martin (Table 1) (http://www.bioinf.org.uk/abs/info.html).

**Table 1:** CDR regions boundaries according to each numbering scheme.

Scheme	Chain type	CDR1 start	CDR1 end	CDR2 start	CDR2 end	CDR3 start	CDR3 end
IMGT	Heavy/light	27	38	56	65	105	117
Kabat	Heavy	31	35	50	65	95	102
	Light	24	34	50	56	89	97
Chothia	Heavy	26	32	52	56	96	101
	Light	26	32	50	52	91	96
Martin	Heavy	26	32	52	56	96	101
	Light	26	32	50	52	91	96

Kabat, Chothia, and Martin schemes perform numbering in a distinct fashion to IMGT by employing indel positions. The indel positions are anchors for insertions and deletions. Insertions are placed after the specified indel residue position and deletions before. Anchor positions for all CDRs are defined in Table 2.

**Table 2:** CDR indel positions for Kabat-derived schemes.

Scheme	Chain type	CDR1	CDR2	CDR3
Kabat	Heavy	35	52	100
	Light	27	52	95
Chothia	Heavy	31	52	100
	Light	30	52	95
Martin	Heavy	31	52	100
	Light	30	52	95

While preparing germline-scheme mappings, we noticed that some alleles do not comply with scheme definitions by having additional insertion places not defined by schemes. We decided to add these insertions; otherwise, such positions would cause a misalignment of germline sequences. This behavior is consistent with the other numbering tools. We numbered the V allele sequences with novel insertions with ANARCI and AbRSA, and both programs added insertions in places not allowed by schemes. Insertion places inferred from germline sequences are defined in Table 3.

**Table 3:** Novel germline-inferred insertion positions in Kabat-like numbering schemes.

Chain type	Position	Schemes	Example
Light	66	Kabat, Chothia	IGLV6–57*01, IGLV5–39*01, IGLV5–45*01
Light	52	Kabat, Chothia	IGLV5–45*01, IGLV4–60*03
Light	68	Martin	IGLV6–57*03, IGLV5–39*01

### Allele matching

The allele matching process consists of two steps: prefiltering used for candidate allele selection and pairwise alignments of the selected allele candidates against query sequence. The candidate with the highest e-value is assigned as the matching allele.

In 2017, Martin Steinegger and Johannes Söding demonstrated significant speed improvements in sequence search over Basic Local Alignment Search Tool (BLAST) [24] by introducing diagonal based “prefiltering” step before the alignment in their Mmseqs2 pipeline [25]. In this work, we implemented this concept for the purpose of fast V, D, J, and C allele matching.

**Figure 4:**
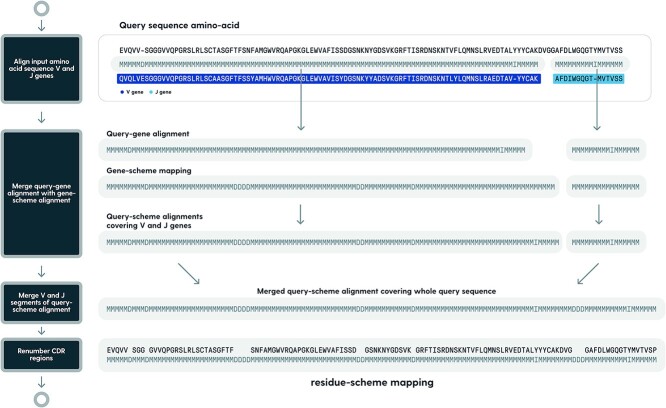
Applying numbering schemes: query amino acid sequence is aligned to germlines to obtain query-germline alignments. This mapping is merged with known germline-scheme mapping for the scheme of choice. This way query-scheme mappings are obtained for V and J segments, which are subsequently joined to create query-scheme mapping covering the whole input sequence. We use “unfolded” CIGAR as the pairwise alignment notation, where “M” denotes positional match between query and target sequence and “I” and “D” denote insertion and deletion on query, respectively.

Prefiltering is a crucial step in the RIOT allele matching pipeline that helps reduce the computational time and resources required for the alignment process. In this step, an index table for target sequences is created. For each *k*-mer occurring in the target gene sequence, one can look up the target’s identifiers and *k*-mer positions. These *k*-mers are then used to identify the best matching allele candidates to the query sequences by calculating diagonal scores. If we construct a coordinate plane in which we denote the *x*-axis as the position in the query sequence and the *y*-axis as the position in the target allele, all *k*-mer matches between the input sequence and target allele can be represented as short diagonal segments starting at coordinates (query position, target position). Consecutive segments can be directly connected to form contiguous sequences of consecutive matching positions. If there is an insertion in the query sequence with respect to the allele sequence, it will be represented as a segment shifted along the *x*-axis. Similarly, a deletion will be represented as a shift along the *y*-axis. Segments with a difference between *x* and *y* positions in consecutive matches greater than the threshold are discarded. Finally, the number of matching residues serves as a coverage (or diagonal) score, which is used further to select the best matching candidates (Fig. 4). By setting a threshold on this score, RIOT filters out sequence pairs that are unlikely to share significant similarity, retaining only the most promising candidates for further processing. This prefiltering step dramatically reduces the number of sequence pairs that need to be considered in the subsequent, more computationally intensive pairwise alignment.

Prefiltering can be fine-tuned on the number of best matches returned, the size of *k*-mers for the index, distance threshold, and sampling threshold. While the sampling threshold and k-mer size impact the number of *k*-mer comparisons, which has a direct influence on prefiltering performance, when it comes to the number of best matches returned—prefiltering execution time does not change itself but this alters the number of pairwise alignments performed downstream. As consecutive *k*-mer matches constitute diagonal construction, the distance threshold is the maximum relative offset between query and target positions for *k*-mer to be included in the diagonal. Finally, the sampling threshold determines the offset between consecutive *k*-mers from the query sequence.

RIOT prefiltering parameters were fine-tuned using a grid search for each allele with the goal of minimizing the number of consecutive alignments while preserving germline alignment accuracy on a sample dataset of human sequences. Ground truth allele assignments were obtained by aligning input sequences to all germline sequences in the database and the alignment with the lowest e-value was selected as the correct germline. All RIOT prefiltering parameters used are summarized in Table 4. To further speed up the allele candidate selection, we used the *ahash* library (https://docs.rs/ahash/latest/ahash/) for rapid hash calculations of *k*-mers in index lookups.

**Table 4:** Prefiltering parameters for alleles. Top *N* is the number of sequences passing prefiltering to subsequent pairwise alignments. K-mer size determines the length of k-mers used in the prefiltering index. Distance threshold is the maximum relative offset in query and target positions between consecutive k-mer matches. Modulo *N* is the sampling threshold determined by offset between consecutive k-mers in the query sequence.

Gene	Locus	Top *N*	K-mer size	Distance threshold	Modulo N
V	All	12	9	13	2
D	IGH	5	5	3	1
J	IGH	5	5	3	2
J	IGK	5	5	7	2
J	IGL	5	5	5	1
C	IGH	5	5	5	2
C	IGK	1	5	3	1
C	IGL	5	3	5	1

After the prefiltering step in the RIOT allele matching pipeline, the remaining candidate sequence pairs proceed to the pairwise alignment stage. This step employs the single instruction multiple data (SIMD)-optimized Smith–Waterman algorithm [21] for the selected query–target sequence pairs. The alignment information is stored as a CIGAR (Compact Idiosyncratic Gapped Alignment Report) string, which is a concise representation of the consecutive mapping operations (match, mismatch, insertion, and deletion) and their counts. Finally, alignment scores are calculated, such as sequence identity or e-value. Resulting alignments are sorted by e-value, so it is easy to pick the best one in the last step. Alignment parameters used in RIOT are the same as defaults in IgBLAST. We found them to produce accurate alignments and, at the same time, similar to what the user base is accustomed to. They are summarized in Table 5.

**Table 5:** Pairwise alignment parameters.

Sequence type	Score	Gap open penalty	Gap extend penalty
Nucleotides	Match: 1; Mismatch: −1	4	1
Amino acids	BLOSUM62	11	1

### Germline database

The Open Germline Receptor Database (OGRDB) [17] is used as a primary source of germline alleles in RIOT. It is a reference database for inferred immune receptor genes that addresses the need for comprehensive germline allele reference sets to interpret AIRR-seq data accurately. Current reference sets are incomplete and under-representative of human and animal population diversity [26]. OGRDB provides V, D, and J allele sequences for humans and mice with supporting evidence. Mouse sequences from all strains were put into a single database and deduplicated. This approach was optimized for the accuracy of the numbering and the scenario where no organism is provided on the input. Since germlines of distinct mouse strains may be redundant (have the same sequence), it may happen that RIOT will provide annotations that are not biologically plausible (e.g. it will mix germlines from two distinct mouse strains). For this reason, we have provided an example of how to use RIOT with a custom database for a specific mouse strain. Human C genes were obtained from National Center for Biotechnology Information (NCBI) [27] and are reused for all species. Allele abundance for gene sequences is presented in Table 6.

**Table 6:** OGRDB-derived internal germline database in RIOT. Allele abundance is given for each locus in human and mouse databases.

Organism	Segment	H	K	L
Human	V	190	62	75
	D	31	–	–
	J	7	7	9
	C	9	1	5
Mouse	V	643	459	7
	D	19	–	–
	J	6	11	7

### Output format

RIOT outputs its annotations in extended AIRR rearrangement format (https://docs.airr-community.org/en/stable/datarep/rearrangements.html). Additional columns that were added are defined in Table 7.

**Table 7:** Additional columns in RIOT output.

Name	Type	Definition
scheme	string	Numbering scheme applied in annotation.
v_frame	enum [0,1,2]	V frame offset from v_alignment_start.
j_frame	enum [0,1,2]	J frame offset from j_alignment_start.
scheme_residue_mapping	json string	Numbering of sequence_alignment_aa.
positional_scheme_mapping	json string	Scheme mapping from sequence_aa (0-based).
additional_validataion_flags	json string	JSON string containing validation flags.

### Availability

RIOT is open-sourced via the following repository: https://github.com/NaturalAntibody/riot_na and is free to use for noncommercial use by noncommercial organizations. An online user interface that allows users to annotate a single sequence is available at: https://riot.naturalantibody.com/. Examples that showcase how to use RIOT and how to build a custom germline database for RIOT are linked in the README file located in the github repository.

## Results

### Feature comparisons of existing tools

We compared RIOT to other tools in two dimensions: supported numbering schemes (Table 8) and annotation possibilities (Table 8).

**Table 8:** Comparison of supported numbering schemes in existing tools.

	AntPack	AbNum	AbRSA	ANARCI	RIOT	IgBLAST
Kabat	Yes	Yes	Yes	Yes	Yes	Yes*
Chothia	No	Yes	Yes	Yes	Yes	No
Martin	Yes	Yes	No	Yes	Yes	No
IMGT	Yes	No	Yes	Yes	Yes	Yes*
Aho	No	No	No	Yes	No	No

^*^IgBlast does not provide residue-level annotations like all other tools.

Considering the supported numbering schemes, RIOT falls behind ANARCI by not supporting the Aho numbering scheme. This was a conscious choice since there are multiple numbering systems, not all listed here, and we chose to implement only those that are commonly used within the industry and academic community. While IgBLAST supports Kabat and IMGT in its annotations, it does not provide residue-level annotations.

Regarding annotation features, we list the ability to handle nucleotide or amino acid formats, germline assignment, isotype assignment, and compatibility with the AIRR format (Table 9). From all presented tools, only RIOT and IgBLAST check all boxes in compared features. IgBLAST support for amino acids is limited as it is able to align only V genes.

**Table 9:** Comparison of supported features of existing antibody numbering tools.

	AntPack	AbNum	AbRSA	ANARCI	RIOT	IgBLAST
Nucleotide annotation	No	No	No	No	Yes	Yes
Amino acid annotation	Yes	Yes	Yes	Yes	Yes	Yes*
Germline assignment	Yes	No	No	Yes	Yes	Yes
Isotype assignment	No	No	No	No	Yes	Yes
AIRR format output	No	No	No	No	Yes	Yes

^*^IgBlast only has limited functionality regarding amino acid annotation as it only aligns V-genes.

### Germline assignment accuracy and speed

To assess the performance of RIOT, we compared it to the leading annotation tools available: IgBLAST, ANARCI, and AntPack. Neither AbNum nor AbRSA were included in the comparisons as they do not provide germline assignment functionality. We constructed two benchmarking datasets: containing nucleotide and amino acid sequences to benchmark IgBLAST and ANARCI + AntPack, respectively. The nucleotide sequence dataset was constructed by downsampling the AbNGS [28] dataset, and the amino acid sequence dataset was derived from therapeutics sequences curated from INN entries [29]. All tools tested were configured to use the same germline database: in the case of IgBLAST, we provided the OGRDB germline database as an input, and, for ANARCI, we constructed HMM profiles from V-J gene pairs gapped to IMGT. The IMGT-gapped OGRDB germlines were also provided to AntPack for consistency. In all cases, we assessed the accuracy of assigning germlines as well as wall-clock running time.

To construct ground-truth datasets in both cases, each test-set sequence was aligned to all of the germline allele sequences using the striped Smith–Waterman algorithm, and the allele with the lowest e-value score was assigned as a ground truth. We evaluated the germline assignment accuracy on the level of genes and alleles. As each experiment was performed 10 times on an 8-core machine, execution wall clock time was measured, and the mean value from 10 distinct runs was used to compare the performance.

IgBLAST achieves comparable germline assignment accuracy to RIOT (Table 10). Our software, however, achieves significant speed improvement over IgBLAST, being more than four times faster. We have tuned the parameters for the sample next-generation sequencing (NGS) sequence dataset; however, when working with highly mutated sequences or sparse germline databases, optimal execution parameters could be different and therefore could be adjusted using grid search, as described in the Allele Matching section.

**Table 10:** Comparison of gene assignment accuracy and execution time for IgBLAST and RIOT. Execution time reported is wall-clock from execution on an 8-core machine.

		RIOT	IgBLAST	Dataset
Gene	V	99.85%	99.88%	NGS sample stratified by bioproject (362 180 sequences) *Homo sapiens*
	J	99.91%	98.84%	
Allele	V	99.83%	99.73%	
	J	99.91%	98.80%	
Execution time 8 cores		00:04:26	00:18:02	

The therapeutics-derived dataset was used to measure the germline assignment accuracy for amino acid–based annotation using ANARCI and AntPack. Because of its small size, the execution time measurements were not reliable. Therefore, to measure the processing efficiency, we constructed another amino acid dataset by translating NGS sequences. Germline amino acid sequences were deduplicated as there may be multiple distinct alleles with the same translation sequence.

RIOT outperforms ANARCI on all metrics (Table 11). As we investigated, the surprisingly low germline assignment accuracy of ANARCI could be attributed to its germline assignment method: iterating over the alignment to select the gene with the highest sequence identity. We advocate for assigning genes using e-values from alignments as we believe that this renders more biologically meaningful results rather than sequence identity alone. Since AntPack has a similar germline annotation scheme as ANARCI, it is outperformed by RIOT on this count. Nevertheless, AntPack achieves a significantly lower run-time of 32 s versus 2 min and 40 s in RIOT.

**Table 11:** Comparison of gene assignment accuracy and execution time for ANARCI, AntPack and RIOT. Execution time reported is wall-clock from execution on an 8-core machine. ^*^AntPack assigns genes only in the IMGT scheme - assignments in other schemes require double numbering. Time measurements show the numbering to the IMGT scheme.

		RIOT	ANARCI	AntPack	Dataset
Gene	V	96.94%	65.42%	80.04	Therapeutics heavy and light separate (1274 sequences) V genes, *Homo sapiens*
	J	97.72%	78.94%	77.99%	
Allele	V	96.86%	62.17%	76.35%	
	J	97.65%	78.60%	77.77%	
Execution time 8 cores		00:02:40	00:18:01	00:00:32	362 180 random sequences from AbNGS

### Numbering accuracy

In order to evaluate the numbering accuracy, a therapeutics amino acids sequence dataset was used. All sequences were numbered with AbNum, AbRSA, ANARCI, AntPack, and RIOT to numbering schemes given in Table 8. Numberings were converted to a common format so that all numbering results have unified insertion notation. Finally, the results from all tools were aligned together, so nonconsensus sequences could be identified. AbRSA numbering results were excluded from comparisons as it caused most conflicts (CDR numbering does not comply with scheme definitions).

### IMGT—ANARCI, AntPack, and Rapid Immunoglobulin Overview Tool comparison

Out of all therapeutics-derived variable regions, there were only 23 cases where tools rendered conflicting numberings. Upon examination of the conflicting numberings, we observed that some of the differences can be attributed to different CDR smoothing heuristics between programs. Additionally, the numbering differs in the sequences that are radically distant from germlines, and, with examined examples, we were unable to determine which tool is more correct or less wrong.

While renumbering CDRs, ANARCI reorganizes residues from the loop, with two flanking positions on each side. IMGT definitions of CDR boundaries are used for this regardless of the selected numbering scheme. In contrast to this, RIOT renumbers CDR residues without flanking positions, from the target scheme loop definition. In edge-case scenarios, this leads to different region assignments of the residues. For example, with the absence of highly conserved tryptophan on IMGT 118, with a deletion on input sequence according to query-germline alignment, RIOT will show missing residue and FWR4 will start on position 119, where ANARCI smoothing logic will “pull” the nearest residue from CDR and place it at position 118. Furthermore, the smoothing logic applied by ANARCI in IMGT space that is translated further to the user-selected schemes can lead to issues in Kabat-like schemes.

### Kabat/Chothia/Martin—AbNum, ANARCI, AntPack, and Rapid Immunoglobulin Overview Tool compared

Of all therapeutic sequences, there were ~200 conflicts in numberings. The majority of conflicts could be attributed to differences in CDR numbering heuristics or AbNum assigning the last residues of light chains to 106A instead of 107 in contrast to the remaining tools.

AbNum has two distinct heuristics for CDR numbering in Kabat-derived schemes. For example, if present, deletions in CDR3 are placed to the left of the scheme-defined position for insertions (as defined in Table 2). On the other hand, deletions in CDR1 are placed to the right of the insertion position. Such an approach was introduced in AbNum as an arbitrary design decision inferred from observed CDR sequence lengths. Reliance on observed commonly incomplete data can lead to nongeneralizable solutions (as in the case of the whole Kabat scheme); therefore, RIOT unifies CDR numbering heuristics so that all CDRs are numbered in the same way—if present—deletions are placed to the left of scheme-defined indel position. We believe such an approach improves the interpretability of the results.

After ignoring differences created as a result of either of the above reasons, a number of conflicting numberings remained. Some of them could be attributed to the fact that in some rare cases, ANARCI truncates the middle of the input sequence for Kabat-like schemes. Furthermore, ANARCI fails to number the sequence when there is a deletion on the J gene segment. On the other hand, AbNum fails to number some of the sequences. Finally, as it was in the case of IMGT, sequences that differ drastically from germlines can have different numberings.

## Discussion

Here, we introduced RIOT, software for high-throughput annotation of nucleotide and amino acid sequences of immunoglobulins with comprehensive germline databases for humans and mice derived from OGRDB [17]. Our evaluations show that RIOT outperforms the leading nucleotide-sequence annotation tool, IgBLAST in performance, and the amino-acid sequence annotation tool, ANARCI in both performance and germline assignment accuracy. Though not as fast as the current leading numbering solution, AntPack, it offers a much wider functionality and precise germline annotations being within the range of its speed.

In large-scale NGS studies, processing speed is crucial due to the massive volume of data generated [15, 28] that then needs to be effectively analyzed [30]. Efficient processing allows for quicker analysis, enabling researchers to handle larger datasets and accelerate the pace of scientific discovery. With a typical processing pipeline consisting of both IgBLAST and ANARCI, RIOT significantly enhances this aspect by offering a performance that is approximately eight times faster, by virtue of using a single tool rather than both of them. This improvement in speed is particularly beneficial in large-scale studies, where the ability to rapidly process and analyze data can lead to faster insights into immune responses and disease mechanisms.

Accurate germline assignment is essential in immunoglobulin analysis as it impacts the reliability of research findings. The accuracy with which immunoglobulin sequences are mapped to their corresponding germline genes affects the interpretation of B-cell development and thus affinity maturation. Precise germline assignment is crucial for understanding immune system behavior, developing effective vaccines, and designing therapies for autoimmune diseases and infections. Lineage tree reconstruction is crucial in the analysis of NGS outputs from Phage Display or immunization that are employed in antibody-based drug discovery [31]. It is essential to recognize that while accurate germline assignment is crucial, it cannot always be achieved by sequence analysis. This limitation arises because multiple alleles can share the same sequence (which is more evident in amino acid sequences with synonymous codons) and because of insertions and deletions in junction regions during V(D)J recombination, which assembles the variable region by joining V, D, and J germline genes. Moreover, the recombination process can result in noncanonical recombination events, such as tandem-fused D genes, making it harder to trace back to the original germline genes. Another major challenge is the extensive somatic hypermutation process that introduces point mutations into the variable region genes, creating *de novo* “foreign” sequences that can significantly diverge from their germline counterparts.

The processing speed of modern numbering tools is a result of using fast sequence aligners and reducing the number of alignments being performed. The fastest solution, AntPack, achieves its performance by doing just three alignments to consensus sequences (H,K,L), by compromising germline assignment accuracy. We advocate for assigning germlines through alignment as we think it renders the most biologically accurate results that might be crucial applications such as germlining-based humanization or lineage tree reconstruction.

While RIOT offers significant advancements in processing speed and efficiency, it is important to recognize its potential limitations. RIOT may produce unexpected results when the genes of the query sequence significantly differ from the closest allele in the RIOT database. This discrepancy can lead to incorrect germline assignments, potentially affecting the accuracy of the analysis. Moreover, RIOT may encounter difficulties in cases of frame-shifting modifications. These modifications alter the translation of the J gene–derived sequence segment, making it challenging to accurately determine the boundaries of the framework region.

Though RIOT can offer improvements over IgBLAST and ANARCI for immunoglobulins, it does not address T-cell receptors like the two pieces of software. By making the software available, we hope that the findings related to immunoglobulins can also be applied to T-cell receptors, advancing the biological study of these molecules as well as their therapeutic applications.

The integrity of germline databases is pivotal to the accuracy and reliability of annotation tools such as RIOT. The ongoing effort to build and maintain comprehensive reference germline databases is critically important [18, 32]. Despite their critical importance, these databases often suffer from gaps in coverage, which can significantly hinder the performance of even the most sophisticated annotation software. The continuous effort to update and expand these databases is essential to capture the vast genetic diversity across different populations [26]. However, this endeavor presents a dilemma: whether to maintain a dynamic, constantly updated database or to freeze a version for consistency. On one hand, keeping the database updated ensures that it reflects the latest understanding of germline genetics, providing more accurate annotations. This approach is particularly beneficial for research focused on novel or rapidly evolving immunological phenomena. On the other hand, freezing a database version guarantees consistency across annotations, which is crucial for large-scale databases where comparability between records is paramount. This consistency is essential for longitudinal studies and for ensuring that annotations remain comparable across consecutive runs. The choice between these two approaches is fundamentally a matter of priorities.

In conclusion, RIOT introduces significant improvements in the analysis of immunoglobulin sequences, particularly in terms of processing speed and integration of functionalities. We believe that RIOT will significantly aid in the analysis of immunoglobulins by providing a unified platform for comparing sequences against a reference germline database.

**Key Points**

- Annotation of immunoglobulin sequences is crucial in large-scale repertoire analysis as well as monoclonal antibody therapy development.
- Annotation should be performed using a deterministic algorithm and a versioned germline database to ensure reliable reproducibility.
- Our novel tool, RIOT, incorporates both amino acid and nucleotide input annotations.
- Annotations are fast with respect to established tools, and they are performed using a single germline database reference.

## Data Availability

RIOT is available at https://github.com/NaturalAntibody/riot_na.
